# Multiple paths to rumination within a network analytical framework

**DOI:** 10.1038/s41598-024-61469-6

**Published:** 2024-05-13

**Authors:** Gerly Tamm, Ernst H. W. Koster, Kristof Hoorelbeke

**Affiliations:** Psychopathology and Affective Neuroscience Lab (PANlab), Department of Experimental Clinical and Health Psychology, Ghent University, Henri Dunantlaan 2, 9000 Gent, Belgium

**Keywords:** Psychology, Depression

## Abstract

Theories of rumination have proposed different psychological factors to place one at risk for repetitive negative thinking. A comprehensive empirical test that captures the most relevant contributors to rumination is lacking. Building on influential self-regulatory and metacognitive frameworks, we modeled how key constructs in this context relate to ruminative thinking. 498 participants completed online questionnaires including indicators of rumination, metacognition, promotion goal orientation, effortful control, and depression. We estimated regularized partial correlation networks to investigate unique associations between the different constructs and followed these analyses up with directed acyclic graphs to identify potential pathways towards rumination. Results demonstrated that: (1) both self-regulatory and metacognitive factors were directly linked to rumination, amongst these were (2) positive beliefs, negative beliefs about uncontrollability and harm, cognitive self-consciousness, depression, effortful control, perfectionism, and (lack of) cognitive confidence, and (3) we identified multiple directed pathways, suggesting three direct contributors to rumination while controlling for the influence of all other variables: diminished effortful control, positive beliefs, and cognitive self-consciousness. This study is the first to comprehensively assess metacognitive and self-regulatory frameworks of rumination in a data-driven manner. Our findings suggest that there are multiple pathways towards rumination, which should be incorporated in clinical case conceptualization of rumination and related disorders.

## Introduction

Rumination is a form of repetitive negative thinking with a focus on negative past experiences and feelings. It is a process that is characterized by perseverative thinking that induces negative feelings and is considered a transdiagnostic risk factor for affective disorders^1,2^. There indeed is substantial empirical evidence showing that rumination is directly linked to clinical depression^3,4^, and suicide ideation^5–7^. In addition to predicting depression, rumination is also a key feature of many other mental health disorders, and serves as a transdiagnostic predictor for general psychological health^8,9^.

The current literature includes a multitude of reviews and research papers that have sought to explain the persistence of repetitive negative thinking. These theories have proposed a wide range of different key mechanisms that capture unique aspects of rumination. The Ruminative Response Style Theory (RRST)^4^ is one of the most influential frameworks for rumination in the context of depression. The RRST conceptualizes depressive rumination as a trait-like passive and unhelpful response style to negative mood, and its potential causes and consequences. Other major theories include the Habit-Goal Framework of Depressive Rumination^10^, the Self-Regulatory Executive Function model (S-REF)^11^, the Impaired Disengagement Hypothesis^12^, the Goal Progress Theory^13^, the Control Theory^14^, and the Self-Regulation Risk Phenotype hypothesis^15^. These theories capture different focal points within the cognitive architecture behind rumination, such as the emphasis on metacognitive factors (e.g.^16–18^), and the extent to which rumination is conceptualized as a type of self-regulation (e.g.^19–21^). Interestingly, each of these theories have garnered extensive empirical support for their claims.

Considering that each of these different frameworks of rumination are supported by a multitude of prospective and experimental studies, it is likely that there are multiple etiological pathways towards rumination. This indicates the need for an integrative framework to best capture the mechanisms behind rumination. Building on the existing literature, a few papers have tried to integrate different aspects of rumination into a comprehensive framework. For instance, one of the more recent theoretical models is the H-EX-A-GO-N model^22^, which is supported by substantial evidence for each of the different components. The model proposes multiple loops between key variables that cause state and trait rumination and contribute to its maintenance. These include habit development, executive control, abstract processing, goal discrepancies, and negative bias (H-EX-A-GO-N).

Such integrative models have some challenges still to be overcome. Comprehensive empirical testing is needed to examine the influence and interplay between different psychological processes. Moreover, there is a considerable overlap between different theoretical constructs (e.g., effortful control as part of metacognition, rumination as a form of self-regulation etc.) which can complicate testing differential predictions from each theoretical model. As to date, comprehensive empirical tests of the relative importance of different processes in rumination are mostly lacking. As a result, unique associations (while controlling for overlap) between the key variables proposed in different theoretical frameworks of rumination remain to be explored. There is a need for a comprehensive examination of these associations on a broader level, to capture the main components of different theories. In the next sections, we will elaborate on central constructs in self-regulatory and metacognitive theories of rumination, and propose a data-driven approach to model and empirically test the interplay and directionality between these key aspects.

### From self-regulation to rumination

Self-regulation is a cognitive ability that is concerned with controlling one’s actions, thoughts, and emotions (e.g.^23,24^), which relies on prefrontal top-down executive processing that regulates the allocation of attention resources to enhance goal-directed behavior^25,26^. These resources are used when people need to modify their behaviors, thoughts, and emotions with an aim to reach personal goals. Effortful control^26^ represents one form of self-regulation^27^ which includes a voluntary recruitment of processes (attentional control, cognitive, and behavioral inhibition) that are most appropriate considering a given situation^28^.

From a self-regulatory perspective, rumination has been conceptualized as an emotion regulation strategy: it is an effortful, controlled, and conscious process that aims to reduce negative feelings but can actually have detrimental effects^21^. This assumes that when people ruminate they allocate their attentional resources towards an internal discrepancy (between ideal and the present state) with an aim to resolve this. However, when self-regulation fails (e.g., when the problem cannot be solved), then the ongoing unresolved discrepancy between goals and the current state continues to trigger and maintain ruminative processes, resulting in sustained negative affect. In this context, the theory of regulatory focus^29^ suggests that there are two types of goals that people may pursue in their everyday lives: prevention (avoidance, safety focused) and promotion (approach, aspiration focused) related goals. Importantly, Jones et al.^30^ concluded that a failure to reach promotion goals predicted rumination and this effect was further amplified by negative mood state. Moreover, rumination is known to increase the detrimental impact of promotion goal failure on depressive symptomatology^31,32^.

Setting extremely high goals is an indicator of perfectionism. Thus, individuals with extremely high expectations for themselves could be at risk for continuous goal discrepancy, and related to this, rumination and depression. Indeed, Olson and Kwon^33^ showed that high levels of perfectionism in combination with brooding and stress lead to depressive symptoms over time. These studies point to the need to consider a specific type of goal failure (i.e., promotion goal failure) in combination with perfectionism as potential key contributors to rumination.

### From rumination to self-regulation

When approached from the other end of the self-regulatory perspective, rumination can further amplify a variety of cognitive symptoms that can be seen in depression, such as impaired problem-solving, diminished cognitive performance, and persistent negative affect (as in RRST^4^). This suggests a direct link between rumination and effortful control (also see^34,35^). For instance, rumination is known to negatively impact performance on behavioral measures of attention^36^. However, not all studies suggest a direct link from rumination to effortful control. Instead, alternative patterns of directionality have been proposed by many researchers (e.g.^37,38^), suggesting that rumination-related cognitive impairments may further increase susceptibility to ruminative responses^39^. In line with this, recent studies suggest beneficial effects of experimental manipulation of cognitive control on rumination, further impacting depression severity (e.g.^40^; for a meta-analysis, see Vander Zwalmen et al.^41^).

### The role of metacognition

From a metacognitive perspective, rumination is conceptualized as a strategy that some people use to cope with distress^42^. The most prominent metacognitive theory, the Self-regulatory Executive Function framework (S-REF^11^), proposes that rumination stems from positive metacognitive beliefs about rumination. Based on these beliefs, rumination is used as a tool for coping and self-regulation. These beliefs can be positive (e.g., “ruminating about the past helps me to prevent future mistakes and failures”^43^), or negative (e.g., “ruminating is uncontrollable and harmful”^44^). A recent meta-analysis showed that both positive and negative beliefs significantly predicted rumination and depression^45^. The S-REF model proposes that these beliefs guide the controlled processing system which relies on execution of voluntary attention, linking it back to self-regulatory views of rumination.

In addition to specific beliefs regarding the usefulness and effects of repetitive negative thinking, other metacognitive aspects have been considered relevant in the context of vulnerability for internalizing disorders. These include the need to control thoughts which refers to the beliefs that one needs to be in control of their thoughts at all times, cognitive confidence which is concerned with beliefs about one’s memory performance, and cognitive self-consciousness which refers to the ability to monitor one’s thoughts^46^. Amongst others, these dysfunctional metacognitive beliefs have been related to reduced effortful control^47^. In addition, Spada et al.^48^ showed that these metacognitive aspects are particularly relevant in predicting depression but not anxiety. Therefore, these aspects should be considered when aiming to comprehensively examine the key processes related to rumination.

### Current study

By using a data-driven exploratory approach, this study aims to examine predictions from the major theories of rumination, each of which place different emphasis on how metacognitive and self-regulatory processes contribute to rumination. For this purpose, we will rely on network analysis and directed acyclic graphs to model complex interrelations and identify potential directed pathways of the variables of interest and rumination.

First, we aimed to model centrality of and unique associations between effortful control, indices of promotion goal focus, metacognition, rumination, and depression. Network analytical approaches are increasingly used in psychopathology research^49^ and are particularly suitable for exploration of interconnections between multiple variables. The network theory of psychiatric disorders assumes that psychiatric symptoms causally interact with each other^49^. The data driven approach is the key element here to test whether the theories of rumination hold up in relation to the empirical data while considered within the network analytical framework. The major advantage of this approach is that it considers a set of key variables as a complex system^49^ in which all possible associations can be included and the most relevant links can be detected by applying a data-driven approach (via pre-defined and rigorously tested algorithms). As such, we will rely on undirected regularized partial correlation network models to clarify the complex interplay between self-regulatory and metacognitive aspects of rumination.

Although providing important insights into the structure of the model and centrality of specific variables, a disadvantage of *undirected* models is that they describe the general structure of associations while ignoring the potential direction of effects. In this context, several mathematical optimization techniques and machine learning approaches have been developed that allow to explore directionality in data obtained from cross-sectional studies. Applying such methods to explore the network structure and (potential) directionality willprovide the most comprehensive overview of unique associations between the variables of interest and rumination. As such, to test the potential *directionality* of these associations, we will—as a second step—apply Bayesian network analysis^50,51^.

This approach was developed and further tested by Pearl and colleagues^52,53^, and has been applied to study psychopathological processes by several researchers^51,54^. Bayesian networks rely on the mathematical principles of causal reasoning^50^ which unifies the three theories of causation: the probabilistic^55^, counterfactual^56^, and the manipulationist approach^57^. As a result, they are represented as *directed acyclic graphs* (DAGs) which allow to identify potential directed associations in a data-driven way and provide a future basis for testing specific hypotheses about causality between the key variables. The DAGs can provide an approximation and hypotheses about how different concepts from different theories are positioned in relation to each other within the cognitive architecture of rumination.

## Methods

### Participants

A total of 504 participants between ages 18 and 85 were recruited via Academic Prolific (www.prolific.co) from the United Kingdom (UK). In addition to the 504 participants who completed the study, 23 decided not to submit their data, and 7 participants were timed out. Out of the 504, 6 participants (5 women, 1 man) were excluded due to incorrect responses to at least one of the check items out of three. Thus, a total of 498 were included into the final sample. All participants were fluent in English. The sample’s sex, age, and ethnicity distributions were representative of the UK’s population based on most recent census data (cf. Supplementary Materials, Table S6).

#### Ethics declaration

All participants gave an informed consent, agreed to participate voluntarily, and their time spent was compensated for data was anonymized. The study was approved by the local ethics committee of the Faculty of Psychology and Educational Science of Ghent University. All research was performed in accordance with regulations (incl. Declaration of Helsinki).

### Self-report questionnaires and key variables

The following questionnaires were used to measure the 12 key variables included in the network model. All questionnaires used have gone through a rigorous validation and have demonstrated adequate reliability. Table S1 in Supplementary Materials includes a more detailed discussion of each of the key variables.

#### Rumination and depression

We measured *rumination* with the Ruminative Response Scale (RRS)^58,59^*.* The full version of the 22-item scale included all three factors (brooding, reflection, and depression). Higher scores indicate more frequent rumination. Each item is rated on a four-point Likert scale from 1 (almost never) to 4 (almost always). The RRS has good internal consistency, validity, and high reliability^60^. In our data, the overall Cronbach’s α was 0.95. We used the summary compound of rumination by adding the scores for the 22 items (as in^61^) to capture all aspects of rumination.

We measured *depression* with the Depression, Anxiety, Stress Scales (DASS-21^62^). The DASS is a 21-item questionnaire that includes three factors: depression, anxiety, and stress. Each item is rated on a four-point Likert scale from 0 (did not apply to me at all) to 3 (applied to me very much, or most of the time). Higher scores indicate more frequent symptomatology. Given the focus of the study, we only included the depression subscale (7 items) into the analysis. DASS-21 has acceptable validity and reliability^63^. In our data, the Cronbach’s α for the depression subscale was very high (α = 0.94). In line with the original instructions, we calculated the depression subscale by adding the scores of all depression items and multiplying this by 2^62^.

#### Promotion focus, promotion goal failure, and perfectionism

We measured *promotion focus* with the Regulatory Focus Questionnaire (RFQ^64^). The RFQ is an 11-item questionnaire that captures two factors: promotion and prevention focus. We included the promotion focus subscale consisting of 6 items into the analysis. Each of the items is rated on a Likert scale from 1 (never or seldom) to 5 (very often). RFQ has acceptable validity and reliability^64^. In our data, the Cronbach’s α for the promotion focus subscale was acceptable (α = 0.70). Higher scores refer to more frequent promotion focus. We obtained the summary score for the promotion focus subscale by calculating the average score of the 6 items^64^.

We measured *promotion goal failure* with the Computerized Selves Task (CS^31^) that includes ratings for six adjectives (personal ideals/goals) reported by the participant. Each item is rated on how far away the person was at that time from their ideals on a 7-point Likert scale ranging from 1 (not at all) to 7 (extremely). We calculated the scores based on the original instructions: average score of the six items. Higher scores indicate a greater discrepancy between ideals and the present self. This task has been used in multiple prior studies to measure discrepancy between the ideal and present self as a proxy for promotion goal failure^65^. In our data, the overall Cronbach’s α was 0.89.

We measured *perfectionism* with the Frost Multidimensional Perfectionism Scale—Brief^66^. This 8-item measure has two factors (strivings and evaluative concerns). It has good reliability and validity^66^. In our data, the overall Cronbach’s α was 0.85. We calculated the compound score for perfectionism by adding all items as suggested by Burgess et al.^66^.

#### Metacognitive beliefs

We measured *positive metacognitive beliefs about rumination* with the Positive Beliefs about Rumination Scale which has good validity and reliability (PBRS^43^). In our data, Cronbach’s α was 0.93. The scale includes 9 items, each item is rated on a four-point Likert scale from 1 (do not agree) to 4 (agree very much). We calculated the overall score based on the original instructions as the sum of all items.

We measured two types of *negative beliefs about rumination* with the Negative Beliefs about Rumination Scale (NBRS^44^). The NBRS (13 items) includes two factors: (1) *negative beliefs about uncontrollability and harmfulness of rumination*, and (2) *negative beliefs about negative social and interpersonal consequences*. Each item is rated on a four-point Likert scale from 1 (do not agree) to 4 (agree very much). The NBRS has good psychometric properties^60^. In our data, the Cronbach’s α for each of the factors were 0.88 and 0.85, respectively. The score of this scale was based on the sum of all items per factor.

Additionally, we measured metacognitive beliefs about the *need to control thoughts, cognitive self-consciousness*, and *(lack of) cognitive confidence* with the Metacognitions Questionnaire (MCQ-30^67^). The MCQ-30 has 30 items that are distributed between five factors (*the* *need to control thoughts, cognitive self-consciousness*, *(lack of) cognitive confidence, negative beliefs about uncontrollability and harm, and positive beliefs*). We only used three factors (*need to control thoughts, cognitive self-consciousness*, and *(lack of)* *cognitive confidence*) in the analysis, since other scales captured the other two factors better (i.e., in the MCQ, positive and negative beliefs about one’s thinking are mainly focused on measuring worry rather than rumination, in contrast to the PBRS and NBRS). Each item is rated on a four-point Likert scale ranging from 1 (do not agree) to 4 (agree very much). The MCQ-30 has acceptable validity and reliability^46^. In our data, the Cronbach’s α for each of the factors were 0.75, 0.85, and 0.90, accordingly. We followed the original instructions for calculations and summed all items for each factor^67^.

#### Effortful control

We measured *effortful control* with the effortful control subscale (EC) from the short version of the Adult Temperament Questionnaire (ATQ-EC^28^). The EC subscale has 19 items that are distributed between three factors: inhibitory control, activation control and attentional control. Each item is rated on a Likert scale from 1 (extremely untrue) to 7 (extremely true). EC is often used separately from the ATQ full version to measure effortful control. Higher scores indicate better effortful control. Its validity and reliability are acceptable^28^. In our data, Cronbach’s α was 0.84. Scores were calculated according to the instructions provided by Evans and Rothbart^28^.

### Procedure

This study was carried out online using Limesurvey and Academic Prolific. Data was collected in November 2022. Participants who registered in Academic Prolific and who fit the criteria (based on UK census data) were automatically invited to the study. Participants could fill in the questionnaires using their laptops, computers, tablets or smartphones.

All participants had to log in to their Academic Prolific account and follow the Limesurvey link to the study. First, they completed the sociodemographic questionnaire which was followed by a fully randomized block of all nine questionnaires. After completing the randomized block, participants completed an additional block of questionnaires with a main focus on trauma (not reported here). The sequence of the two blocks was fixed. In addition to the questionnaires, three attention check questions were used to check for careless responding. Median study completion time was 27 min. After completion of both blocks, participants were reimbursed via Academic Prolific platform.

### Data analysis

All data analyses were carried out in RStudio (2023.03.0 Build 386) with R version 4.2.3. Descriptive statistical analysis included arithmetic mean, standard deviation, and visual inspection of the data (histograms and scatter plots). Summarized data and R code are available here: https://osf.io/6zqmd/.

#### Data checks

There were no missing data. After visual and statistical inspection of data distributions we noted that all variables deviated somewhat from the Normal distribution. In order to improve normality and to ease the assumption for Gaussian networks^68^, we used the nonparanormal transformation, as suggested by Epskamp et al.^69^, by applying *huge.npn()* from *huge* package^70^.

We used the *goldbricker()* function from *networktools* package^71^ to check for collinearity between the different variables. We relied on the method proposed by Hittner et al.^72^ to identify highly correlated node pairs (*r* ≥ 0.5) showing similar correlation patterns (≤ 25% unique associations, *α* = 0.05), suggesting that these might measure the same underlying construct based upon which they could be revised. In our dataset, based on tests for collinearity, no reductions to the selected node set were needed.

#### Undirected networks

First, we estimated a *Gaussian Graphical Model* (GGM), also known as *regularized partial correlation networks* or *Markov Random Fields*^49,73^, to explore the interconnections (i.e., edges) between the variables of interest (i.e., nodes; for an overview of included variables, see Table S1 in Supplementary Materials). Partial correlations between nodes (i.e., variables) provide an estimate of unique variation captured by every node while considering all variation (all other nodes) within the network. We included 12 nodes (see Table S1 in Supplementary Materials): rumination (RUM), effortful control (EC), cognitive confidence (CC), positive beliefs about rumination (PB), cognitive self-consciousness (M-SC), need for control of thoughts (M-NC), beliefs about uncontrollability and harmfulness of rumination (NB-U), beliefs about negative social and interpersonal consequences of rumination (NB-S), promotion focus (PRO), perfectionism (PER), promotion goal failure (PGF), and depression (DEP).

#### Network estimation and visualization

The partial correlation matrix was calculated with *cor_auto()* from *qgraph* (based on Lavaan function lavCor^74^) and was used as input for network estimation. We estimated the undirected networks with *qgraph()* from the *qgraph* package^75^. To remove spurious edges (i.e., false positives) from the network, it is recommended to use statistical regularization methods. For that purpose, we used Least Absolute Shrinkage and Selection Operator regularization (gLASSO^76^) from *glasso* package^77^, which is a modified and faster version of the regular LASSO^78^. Often, combined with Extended Bayesian Information Criterion (EBIC^79^), it is used to identify the optimal model, particularly in moderately large samples^69^.

In an exploratory context of many nodes and hundreds of potential edges, gLASSO limits the number of edges by setting potentially spurious (smaller) edges equal to zero. This results in more realistic, and sparser models. gLASSO uses a tuning parameter lambda (λ) which controls the level of sparsity. We used the default settings for λ in *qgraph* package^80^. We used additional thresholding (before EBIC) as implemented in *qgraph* which removed all elements in the inverse variance–covariance matrix that were below a theoretical threshold^81^ to ensure high specificity. Also, we set additional limits to the EBIC model selection procedure by setting the hyperparameter gamma (γ) to 0.5. Gamma is typically set between 0 and 0.5^82^ while 0.5 has been recommended as it reflects a more conservative approach^73,82^.

Undirected networks are visualized using circles (nodes, representing variables) and lines (edges, representing unique associations) to connect the circles. Red/dashed lines refer to negative edges (negative association between the two variables), and blue/full lines refer to positive associations. Line thickness corresponds with the relative weight of the edge (i.e., regularized partial correlation) between the two nodes while considering all other nodes in the model. The Fruchterman-Reingold algorithm^83^ was used, which aims to position more influential nodes in the center of the model.

#### Centrality and predictability

Centrality indices are used to describe how well nodes are connected to other nodes in the network. Strength centrality (i.e., the sum of absolute edge weights of edges connected to that node) was used to identify the most well-connected nodes in the model as an indicator of relative importance. Strength centrality has shown to be one of the most reliable centrality indices^84^. In addition, node predictability is relevant when considering the practical prediction value of the model^85^. Nodewise predictability quantifies how well each node was predicted by all other nodes in the model: how much variance of a node can be explained by the edges connected to that node. It is also a marker for potential influence within that network. High predictability of nodes suggests that the model includes relevant factors. Predictability was estimated with the *qgraph* package (version 1.9.8), and plotted using circular pie-charts around the nodes. The colored areas (here, we used black) indicate the percentage of variance explained by the edges that are connected to that particular node.

#### Accuracy and stability

Estimating accuracy and stability of the network models is crucial to demonstrate the robustness of findings^73^. We tested the accuracy of edge weights, and stability of node strengths with the three steps outlined by Epskamp et al.^69^: (1) First, we calculated bootstrapped confidence intervals for edge weights using a non-parametric bootstrap. We used the *bootnet()* function from *bootnet* package^86^ with *nBoots* set to 1000 and *plot* function to model sampling variability in edge weights; (2) Next, we investigated the stability of the obtained order of node strengths using the case-dropping subset bootstrap procedure^69^. This allows to investigate to what extent the order of nodes in terms of strength centrality remains stable when re-estimating the network model in subsets of the sample. In particular, correlation stability refers to the maximum percentage of cases that can be dropped so that the correlation between the original and the bootstrapped networks is above 0.7 for 95% of the cases (as recommended by Epskamp et al.^69^; (3) Finally, the procedure included bootstrapped difference tests between all paired edges, and between all nodes.

#### Directed acyclic graphs

In the second part of the data analysis, we aimed to explore the potential directed relationships between all nodes that have been included in the undirected network model (see Table S1 in Supplementary Materials). For this purpose we used *Bayesian networks* (also known as *directed acyclic graphs*, DAGs)^51,87^. Gaussian DAGs have parametric assumptions such as linear Gaussian distribution with the normal-Wishart prior^68^. To satisfy this assumption, we relied on the transformed data (section Data Imputation, and Transformation).

We estimated the structure of a DAG with a constraint-based modern version of the *Parent–Child algorithm: PC stable*^88^*.* We used the *pc.stable()* function from the *bnlearn* package^89^. PC stable is developed from the inductive causation^90,91^, and it is concerned with finding best matches for each potential directed pair of nodes: i.e., a parent (“from”) and child (“to”) pair. PC stable learns the directed structure from the data as follows^51^: (1) it estimates a network model including pairwise connections between all nodes; (2) then removes edges between conditionally independent pairs of nodes, and; (3) assigns directions (*arcs*—i.e., directed edges in DAGs^51^) to all edges by starting from colliders (also known as v-structures, where two disconnected nodes cause the third node); (4) To ensure the robustness of the DAG, we then continued with bootstrapping procedures to exclude unstable edges. For this purpose, we applied the PC stable algorithm to the data 1000 times, and used 0.85 as a threshold for edge strength and 0.5 as a threshold for minimum direction (the percentage of networks having the same directions for that particular edge) to trim down the number of potential connections between nodes^51^.

We considered the directed model with thresholding as the final model and based our conclusions on the results of this final step. We plotted the directed network with and without thresholding. In these plots, circles represent nodes, and arrows represent potential directed relationships between the nodes. In the plot that includes all potential directions, edge thickness represents the percentage of times the edge was present in this particular direction in the set of 1000 bootstrapped solutions. To understand the meaning of the predicted directed associations between each pair of nodes, we conducted pair-wise regression analyses to test how nodes were connected. That is, we checked whether there was a positive or negative beta value.

## Results

Sample size characteristics and descriptive statistics of all variables included into the network analysis are described in Table 1. Further details about each of the variables can be found in Table S1 in Supplementary Materials. Pearson correlations between all variables are described in Supplementary Materials, Table S2.

**Table 1:** Descriptive statistics of the sample (n = 498) and key variables.

Sociodemographic characteristics		
Age (mean, SD)	46 years (SD = 16 years)	
Gender (n)	Men (237), Women (255), Other (5), Prefer not to respond (1)	
Ethnicity (n)	White (431), Black (18), Asian (35), Mixed (11), Other (3)	
Education (n)	Graduate (85), Undergraduate (196), Vocational (82), Secondary or less (135)	
Employment (n)	Employed (340), Student (29), Retired or on leave (106), Prefer not to respond (23)	

Key variables^a^	Mean	SD
Promotion goal failure (PGF)	3.17	1.69
Effortful control (EC)	4.57	0.89
(Lack of) cognitive confidence (M-CC)	10.64	4.47
Cognitive self-consciousness (M-SC)	14.33	4.41
Need for control (M–N)	11.10	3.67
Positive beliefs about rumination (PB)	18.14	6.61
Negative beliefs: uncontrollability and harm (NB-U)	13.97	5.41
Negative beliefs: social consequences (NB-S)	6.79	2.79
Promotion focus (PRO)	3.27	0.69
Rumination (RRS)	44.25	14.62
Depression (DEP)	11.72	11.46
Perfectionism (PER)	22.84	6.53

^a^All key variables were included into network analysis.

### Undirected networks

**Figure 1:**
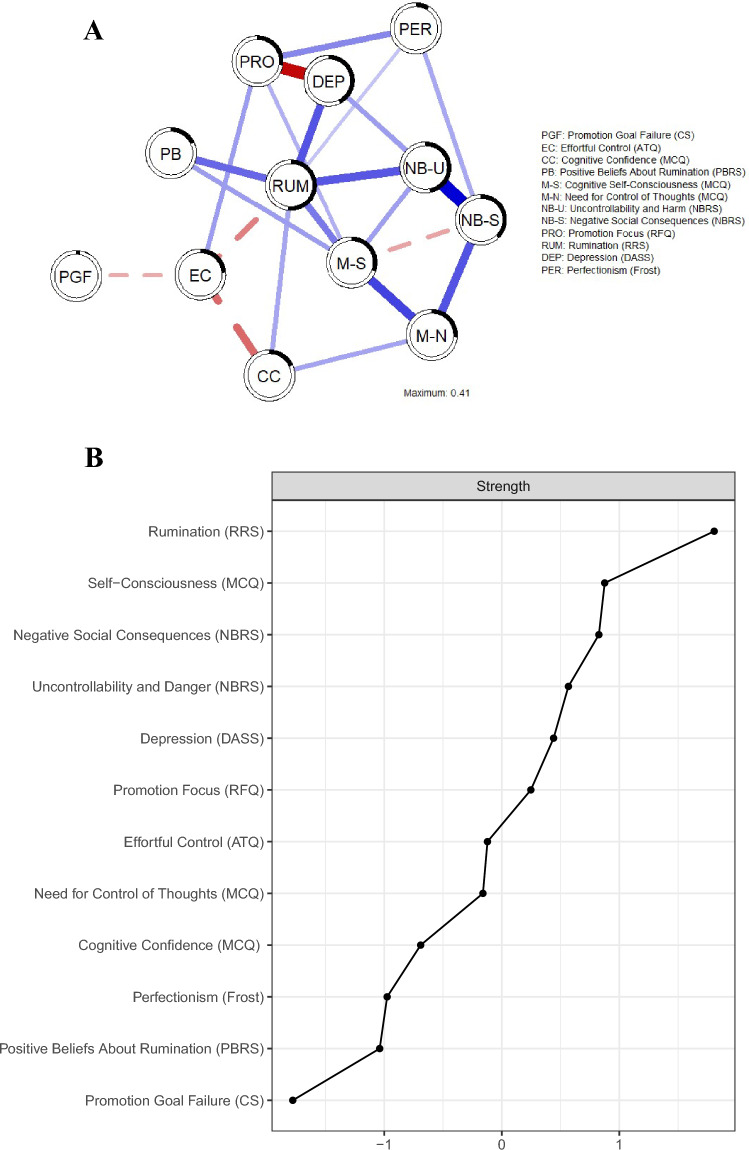
GGM. (**A**) Undirected network model. Blue/full lines indicate unique positive associations and red/dashed lines indicate negative associations between the variables (nodes). Black circles around the nodes indicate node predictability. (**B**) Centrality of the nodes: Strength. Higher strength indicates relative importance of that node in the network model.

First, we estimated the GGM (Fig. 1A). The centrality index (strength) is visualized in Fig. 1B. Additional accuracy and stability checks show that the obtained network model was relatively accurate and stable (Supplementary Materials, Figs. S1–S3; strength correlation stability = 0.67). The edge weight matrix for the GGM can be found in Supplementary Materials, Table S3.

As expected, rumination was ranked first in terms of Strength centrality (Fig. 1B), given the selection of nodes based on theoretical frameworks of rumination. This suggested that rumination was among the most influential variables in this network. When considering all potential associations between the nodes, the model showed that rumination (RUM) was directly and positively associated with depression (DEP), positive beliefs about rumination (PBR), negative beliefs about uncontrollability and harmfulness of rumination (M-BU), cognitive self-consciousness (M-SC), (lack of) cognitive confidence (CC), and perfectionism (PER); furthermore, rumination (RUM) was directly negatively associated with effortful control (EC).

There was a strong direct connection between depression and promotion focus (negative association), which was also related to effortful control (positive association) and perfectionism (positive association). In addition, negative beliefs about social consequences also showed relatively strong connections with  negative beliefs about uncontrollability and harm (positive association) and the need for control of thoughts (positive association), the latter also being connected with cognitive self-consciousness (positive association) and (lack of) cognitive confidence (positive association). The least central node in this model was promotion goal failure, which was directly associated with effortful control only (more feelings of failure were associated with lower effortful control).

We estimated predictability (R^2^) of the nodes from the GGM model. Rumination had the highest predictability, and promotion goal failure had the lowest predictability. Mean node predictability of the conservative model that included additional thresholding was 0.29, which means that on average 29% of variance in nodes was explained by surrounding nodes. Predictability of rumination was 52% (with additional thresholding).

In sum, these findings indicate tha—while controlling for the influence of all other variables in the model—multiple constructs from metacognitive and self-regulatory theories share a direct association with rumination. Together, these variables predict a significant amount of variability (52%) in rumination within the sample.

### Directed acyclic graphs

**Figure 2:**
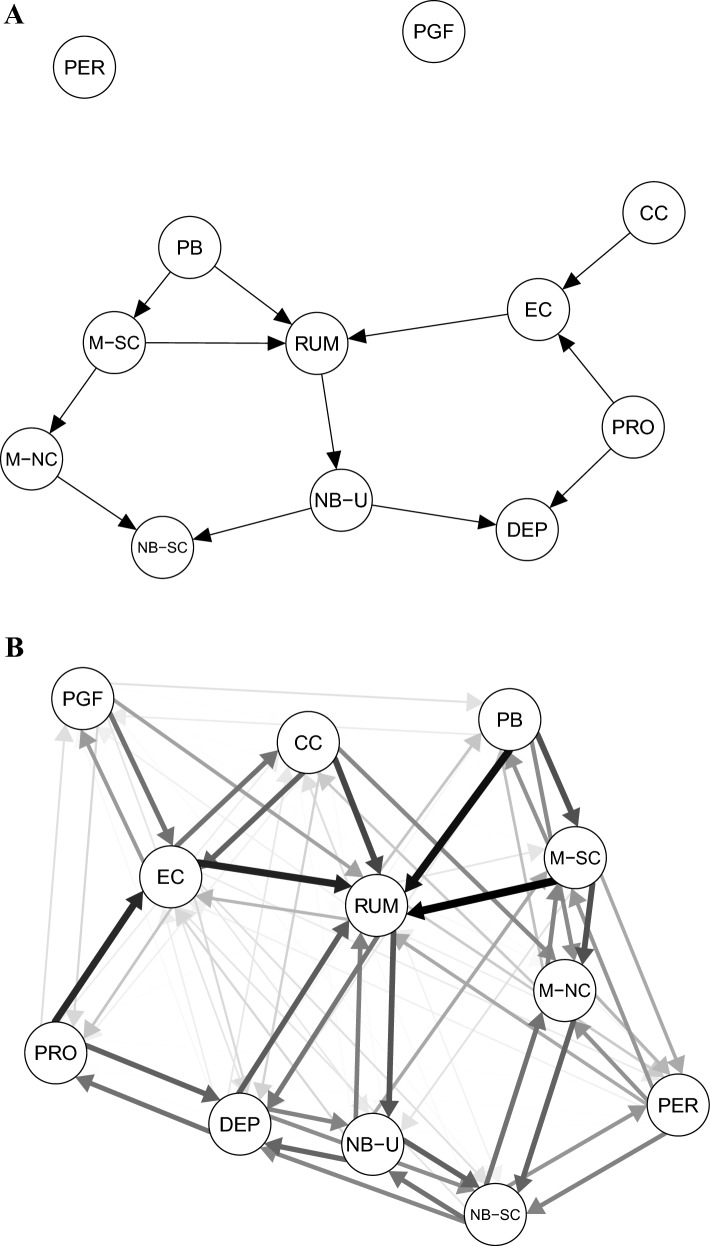
DAG. (**A**) Averaged Directed Acyclic Graph. Arrows indicate potential causal associations between the variables (nodes). (**B**) Bootstrapping Results. The graph illustrates all associations that were present in the data before applying the threshold to trim down the most likely and most relevant associations which are presented on panel (**A**). *PGF* promotion goal failure, *EC* effortful control, *CC* (lack of) cognitive confidence, *PB* positive beliefs about rumination, *M-SC* cognitive self-consciousness, *M-NC* need for control, *NB-U* negative beliefs about uncontrollability and harm, *NB-SC* negative beliefs about social consequences, *PRO* promotion focus, *RUM* rumination, *DEP* depression, *PER* perfectionism.

Next, we estimated DAGs with the constraint based PC stable algorithm to identify potential directed pathways. Figure 2A illustrates the averaged DAG after bootstrapping, and applying the threshold (0.85) for edges. The obtained model suggests several potential pathways towards rumination. First, the obtained pathways suggest that lower cognitive confidence (i.e., more "lack of cognitive confidence") may lead to reduced effortful control, increasing the likelihood of rumination (for corresponding regression coefficients, see Supplementary Materials, Table S4).

In parallel to that, the results suggest that stronger promotion focus results in higher levels of effortful control, reflecting a potential protective effect, and lower depressive symptomatology. Positive beliefs about rumination increase the likelihood of ruminating both directly as well as indirectly via increased cognitive self-consciousness. Rumination is related to further activation of negative beliefs about uncontrollability and harm, which feeds into depression and negative beliefs about social consequences. Through need for control of thoughts, activation of cognitive self-consciousness also feeds into negative beliefs about social consequences of rumination. Perfectionism and promotion goal failure were unconnected in the model.

Figure 2B contains the unthresholded DAG including all estimated directions with edge/arc probabilities after 1000 bootstraps (see Supplementary Materials, Table S5 for bootstrap results), which allows evaluation of the robustness of the obtained edge directions. Although the PC stable algorithm identified several edges/arcs that were relatively stable, in some cases it was difficult for the algorithm to determine the directionality between node pairs. For instance, an edge from rumination to depression was present in 46% of the 1000 bootstrapped samples, and from depression to rumination in 54% of the bootstrapped samples. In the final averaged DAG, no direct connection between rumination and depression was included. This may imply that the algorithm could not determine directionality based on this data due to the bidirectional nature of these relations in combination with the conservative threshold (0.85) for strength.

In sum, the averaged DAG suggests effortful control, positive beliefs about rumination and cognitive self-consciousness to contribute to rumination directly, further spiraling into activation of negative metacognitions regarding rumination, and related to this, depressive symptomatology. Promotion goal focus indirectly impacted rumination via effortful control.

## Discussion

This study aimed to explore the interplay between rumination and related factors proposed in major theories of rumination. We applied a data-driven network analytical approach to examine unique associations as well as potential directed pathways between rumination, metacognitive beliefs and abilities, effortful control, depression, perfectionism, promotion focus, and promotion goal failure. The network models suggested that there were multiple paths to rumination. These results are the first to show that the major theories of rumination that put different emphasis on metacognitive and self-regulatory aspects of rumination complement each other as demonstrated by a comprehensive data-driven model. The main results that emerged from the data driven analysis are in line with more recent theories of rumination, such as the S-REF^16^ as well as with the H-EX-A-GO-N model^22^, and provide empirical background for the therapeutic interventions that are built upon those theories (e.g., metacognitive therapy). We will elaborate on the main findings, clinical implications, limitations, and future directions below.

### Undirected network

First, the undirected network model included a set of variables based on major theories of rumination, which had not yet been incorporated and modelled in a single study. To investigate patterns of unique association between these different constructs, we estimated a GGM.

The undirected network model (Fig. 1A) indicated that seven key variables were directly linked to rumination: (1) depression; (2) negative beliefs about rumination (uncontrollability and harm); (3) positive beliefs about rumination; (4) cognitive self-consciousness; (5) effortful control; (6) perfectionism, and; (7) cognitive confidence. Some of these associations were weaker than others. For example, perfectionism had a significantly weaker direct association with rumination than most other variables mentioned above (Supplementary Materials, Fig. S3A). Moreover, effortful control was negatively associated with rumination, whereas the other aspects were positively associated with rumination. Together, these variables explained 52% of observed variance in rumination in the thresholded model, suggesting that our model contained most central constructs in this context.

Overall, our findings bring together and support self-regulatory (e.g.^12–14,19^, as well as metacognitive perspectives on rumination^17^. Moreover, our results are in line with recent empirical findings that suggest that positive metacognitive beliefs and diminished attentional control both independently contribute to rumination^92^. Importantly, our results expand this notion by adding that there are multiple roads to rumination: several metacognitive aspects, as well as negative and positive beliefs about rumination, and perfectionism share unique and direct associations with rumination.

In addition to the direct links with rumination mentioned above, we observed several indirect links within the network. Promotion goal failure, promotion focus, need for control of thoughts, and negative beliefs about social consequences were part of the rumination network but these processes were not directly linked to higher levels of rumination. Instead they contributed to rumination in an indirect manner. For instance, promotion focus was only connected to rumination through other nodes, among which depression and effortful control. This suggests that people who are focused on their ideals and goals (promotion focused), may report less depressive symptoms, and vice versa. Similarly, promotion focus was related to increased effortful control, in turn linking promotion focus to reduced rumination, and vice versa. These findings are in accordance with Jones et al.^30^ who showed that experimental manipulation of promotion focus (participants were prompted to think about past promotion goal failures) was associated with rumination and negative affect.

Interestingly, effortful control was the only variable linking promotion goal failure with rumination and other constructs, including promotion focus, in the model. Our result suggests that people who experience a stronger discrepancy between their ideal and present self, report less effortful control, which correlates with rumination. This is partially in accordance with the framework outlined in the H-EX-A-GO-N model^22^ which refers to a direct link from goal discrepancy to rumination but also notes that executive functions can influence both rumination and perception of goal discrepancy. One interpretation of this finding is that in order to process discrepancies between the ideal and the present self, one needs to effortfully direct attention to the problem with an aim to solve this. From a self-regulatory perspective, it is generally accepted that promotion goal failure triggers rumination^14,22^, and it is supported by experimental findings showing that cueing unresolved goals results in more rumination than cueing a resolved goal, especially in habitual ruminators^93^. However, our model adds the potential role of effortful control in linking both constructs. As such, when a person is trying to resolve the discrepancy between the ideal and the present self by applying effortful control, then attention directed at trying to find a solution or an explanation to the discrepancy may reduce the ability to focus and work towards a solution. Although undirected, these results suggest that effortful control may play a crucial role in maintaining rumination.

The undirected network model included other variables that were indirectly linked to rumination. Interestingly, metacognitive beliefs about the need for control of thoughts, and negative social consequences were strongly connected but differentially linked to rumination. Rumination and need for control were connected through cognitive self-consciousness, suggesting that people who believe that one needs to be in control of their thoughts are likely to report being more self-conscious, which is linked to more rumination, and vice versa. However, our model suggests that people who believe that rumination has negative social consequences are also more likely to believe that rumination is uncontrollable, which correlates with more rumination (and vice versa). These multiple links to rumination further demonstrate the heterogeneous and complex etiology of rumination.

### Causal inference

After obtaining the network structure, our second goal was to identify potential directed pathways between the variables of interest by applying Bayesian network analysis to the data. Causal inference methods have been used for decades, but they are relatively new in psychopathology research^94^. DAGs are useful and low-cost tools to generate hypotheses about directed effects which can later be tested with experiments.

First, by visual inspection we noticed that the averaged DAG resulted in two interconnected sets of variables. One included several metacognitive variables, and the other one included the main variables from self-regulatory theories, in addition to cognitive confidence (Fig. 2A). Second, the model depicted three potentially directed pathways to rumination: One potential directed pathway suggests a central role for effortful control, where lower cognitive confidence may lead to reduced effortful control, increasing the likelihood of rumination (for corresponding regression coefficients, see Supplementary Materials, Table S4). This finding is consistent with the metacognitive approach, which implies that changes in rumination can be induced by modifying top-down metacognitive processes and beliefs, and by strengthening attentional control (away from self-focused attention)^16,17^. Furthermore, the model suggested that effortful control also receives input from promotion focus, potentially resulting in less rumination. The second directed pathway involves positive beliefs about rumination directly leading to more rumination. The third pathway involves positive beliefs about rumination leading to higher cognitive self-consciousness which leads to more rumination. This suggests that these three aspects are relevant in triggering or maintaining ruminative thinking. Importantly, our model also contained a pathway indicating that rumination may further result in activation of negative beliefs about repetitive negative thinking, and subsequent depressive symptomatology.

We estimated the averaged DAG using the widely implemented PC stable algorithm^88^, and relied on bootstrapping and thresholding procedures to obtain an accurate and stable model. In addition, we applied an alternative model estimation algorithm to test the robustness of the model (the hybrid Min–Max Hill Climbing algorithm, MMHC^95^; Supplementary Materials, Fig. S4). These different algorithms capture different aspects of the data^96^. In general, the obtained DAG model (Fig. S4) was highly comparable in terms of observed associations linking the key variables of interest with rumination, with the exception of one additional direct link from rumination to depression. This is also in line with the findings obtained from the undirected network model (Fig. 1A). The directions, however, were variable when comparing the two DAGs. This difference could be attributed to the potential reciprocal relationships between the variables of interest, which is also consistent with the findings from the bootstrapping procedure (Fig. 2B). In the context of proximal risk factors for repetitive negative thinking, many of the observed associations can, indeed, be bidirectional in nature as also suggested, for example, by the H-EX-A-GO-N model^22^ as well as outlined in the metacognitive models^16,18^. Moreover, the multi-node-loop hypothesis is further supported by empirical findings from another network study which focused on describing the associations between state rumination, self-criticism and behavioral executive control^97^. This is in accordance with the metacognitive models that suggest that the vicious cycles between beliefs about rumination, depression, metacognitive efficiency and confidence may be the underlying cause for persistence of depression^16,42^. Systematic feedback loops have also been proposed by other theories, including a broader system’s dynamic framework that includes biological, cognitive, as well as societal and environmental factors that can reinforce rumination and depression^98^. It is important to note that these theories do not exclude the role of causal factors in triggering rumination and depression. Instead, they emphasize the need to explore the causal pathways and entry points that lead into the vicious feedback loop of rumination and depression. This can be achieved via exploring the data with Bayesian networks, followed up by experimental and simulation studies.

### Clinical implications

These data-driven results bring together and affirm several predictions from prior major theories of rumination and depression, and potential clinical implications. The results confirm the proposition from the metacognitive theory for depression^44^ which predicts that positive beliefs about rumination can directly initiate rumination. This suggests that in clinical case conceptualization, individual metacognitive abilities and beliefs need to be considered. Indeed, belief modification has proven to benefit many but not all patients who experience depressive rumination. Self-regulatory theories (e.g.^13,14^) emphasize the ideal-actual self-discrepancy as the core trigger and maintaining factor for rumination, and suggest that the ability to shift attention away from the negative thoughts is the key intervention point. In the current study, the direct association between effortful control and rumination suggested that modifying attentional control by training attentional disengagement from ideals/goals, or from the negative thought content could result in diminished rumination, as supported by some experiments (e.g.^93^). The S-REF model^17^ provides a more comprehensive framework and emphasizes that the specific complex pattern of thinking called the “Cognitive Attentional Syndrome” causes and maintains psychopathology; the syndrome includes self-focused attention, attentional biases, worry, and rumination. It assumes that multiple cognitive processes simultaneously contribute to psychopathology. Our model indeed shows that attentional processing, beliefs about rumination, and other metacognitive aspects all contribute to rumination. Our results do not provide a direct empirical proof for the effectiveness of any psychological intervention but the model outlines a set of key variables, and associations to rumination, to be considered in case conceptualization. The results emphasize the relevance to explore alternative pathways to rumination in clinical case conceptualization.

### Limitations and future directions

This study is the first to provide a data-driven test of unique associations between key constructs from self-regulatory and metacognitive frameworks of rumination, as well as to explore potential directed pathways between these constructs. Similar to other network analyses in rumination research^97^, we acknowledge that network analysis is a correlational method that has some interpretational limits. For instance, while LASSO regularization helps to avoid false positive results, it increases the chances for missing potential associations that could be relevant in explaining the underlying mechanisms. Importantly, in the current study, the variables included into the model with additional thresholding explained up to 52% of the variation in rumination, which suggests that the model captured a large proportion of the mechanism behind rumination. Moreover, we used a moderately large population representative sample which reaffirms the stability of the obtained results.

To identify potential directed pathways, we relied on DAGs. Although often used in psychopathology research, DAGs assume *acyclicity* of the underlying mechanism. However, many psychological mechanisms include cycles in which the outcome variable can affect the input (e.g., A causes B, B causes C, C causes A). Importantly, it has been demonstrated that even if the assumption for acyclicity is not met, the retrieved set of directions between variables is the *most likely set of causal effects*^99^. Thus, the main value of DAGs in psychopathology research is that they provide a data-driven view on potential directed effects. However, this approach should be considered as strictly exploratory and hypothesis generating. The results should be interpreted with caution. These potential directed pathways need to be confirmed through prospective (e.g., studies relying on experience sampling methodology) or experimental research.

The current study relied on a representative sample for the adult UK population, which is informative for the process of rumination and related factors in the general population. However, we cannot exclude that other mechanisms may be involved in clinical or specific at-risk populations. As such, for future research, it would be interesting to extend the investigation of the role of self-regulatory and metacognitive factors in rumination, and how this adds to mental health complaints, to (sub)clinical samples (e.g., individuals suffering from major depression).

In addition, differential pathways may be observed when focusing on other types of repetitive negative thinking and related factors (e.g., worry in the context of anxiety). Here, we did not include all aspects from prior theories for technical reasons, such as multicollinearity, and for theoretical consideration because we aimed to focus on depression related variables rather than anxiety. One important aspect that should be considered in the future studies is the habitual aspect of rumination. The H-EX-A-GO-N model suggests that habitual rumination is relevant in the maintenance of psychopathology. Here, within this cross-sectional study we did not aim to separate this particular aspect from the global measure of rumination (RRS) but in future studies it could be advisable to explore how rumination develops and persist in daily life by using suitable prospective and experimental designs to capture the habitual component. Other important aspects that could be explored further include abstract thinking, subtypes of rumination (brooding and reflection), and personality characteristics that associate with perfectionism (e.g., neuroticism) which could further elucidate the complex system behind rumination.

## Conclusions

This study explored the complex interrelations between rumination and central constructs from related self-regulatory and metacognitive frameworks in a large adult population sample. The data-driven approach revealed that metacognitive and self-regulatory aspects complement one another by adding unique variance to explain rumination. We estimated undirected and directed network models, which suggested that there are multiple paths to rumination. The three main potential causes for rumination included: positive beliefs about rumination, effortful control, and cognitive self-consciousness. While these findings provide considerable support for the therapeutic approaches that combine the metacognitive belief modification, and attention control training in treatment of depression, the causality of these processes needs further investigation. These results specify hypotheses about the potential causal mechanisms for rumination to be tested in prospective or experimental studies. Finally, these results have the potential to help develop novel therapeutic approaches where, based on the current results, it would seem wise to target multiple factors driving rumination^1^.

### Supplementary Information

Supplementary Information.

## Data Availability

Summarized data and R code are available here: https://osf.io/6zqmd/.
