# Supplementary material for: Multiple paths to rumination within a network analytical framework
Source: Sci Rep. 2024 May 13;14:10874. doi: 10.1038/s41598-024-61469-6 (PMC11091050; doi:10.1038/s41598-024-61469-6)
Supplement: Supplementary file 1 — Supplementary Information. [file 41598_2024_61469_MOESM1_ESM.pdf]

Supplementary Materials

Table S1. List of Key Variables (Nodes)

Node	Label	Description	Range and meaning
Rumination, depression and effortful control			
RUM	Rumination	Summary score of the Ruminative Response Scale. RRS measures the tendency to ruminate about one's negative feelings, and its potential sources. (Example item: *"How often do you …. go someplace alone to think about your feelings"*).	22 – 88<br>Higher scores refer to more rumination.
DEP	Depression	Summary score of the Depression subscale of the Depression Anxiety and Stress Scale. Depression subscale measures the level of depression related negative emotional symptoms on a continuum. (Example item: *"Indicate how much over the past week did this apply to you …. I felt I wasn't worth much as a person").*	0 – 42<br>Higher scores refer to greater depressive symptom severity.
EC	Effortful control	Summary score of the Effortful Control subscale from the Adult Temperament Questionnaire. EC reflects self-regulation abilities in various situations. It includes three subscales: inhibitory control, attention control, and activation control. (Example item: "*It is often hard for me to alternate between two different tasks*")	1 – 7<br>Higher scores refer to better self-regulation.
Goal discrepancy related aspects			
PRO	Promotion focus	Summary score of the Promotion factor from the Regulatory Focus Questionnaire. Promotion focus reflects a tendency to focus on hopes and accomplishments. (Example item: "*How often have you accomplished things that got you "psyched" to work even harder?").*	1 – 5<br>Higher scores refer to more promotion centered regulatory focus.

PGF	Promotion goal failure	Summary score of the Computerized Selves questionnaire. Promotion goal failure indicates the level of discrepancy between the ideal self and perceived present self as described by a list of subjectively chosen adjectives. (Example item: *"Insert an adjective (e.g., "happy") that describes you; what kind of person would you ideally like to be. Indicate how far away are you from this goal (i.e. "happy") at present."*)	0 – 7<br>Higher scores refer to <u>more discrepancy between ideal goals and perceived reality.</u>
PER	Perfectionism	Summary score of the Frost Perfectionism Scale. Perfectionism is a personality trait that includes two types of beliefs about oneself: strivings and evaluative concerns. (Example item: "*If I fail at work/school, I am a failure as a person*.")	4 – 20<br>Higher scores refer to <u>greater perfectionism</u>.
Metacognitive aspects			
CC	(Lack of) cognitive confidence	Summary score of the Cognitive Confidence factor from the Metacognitions Questionnaire. CC captures lack of confidence about one's memory performance. (Example item: *"I do not trust my memory*").	6 - 24<br>Higher scores refer to <u>lower confidence</u> about one's memory.
M-SC	Cognitive self-consciousness	Summary score of the Cognitive Self-Consciousness factor from the Metacognitions Questionnaire. SC captures awareness and monitoring of one's thinking. (Example item: *"I am constantly aware of my thinking").*	6 – 24<br>Higher scores refer to <u>more self-monitoring</u>.
M-NC	Need for control of thoughts	Summary score of the Need to Control Thoughts factor from the Metacognitions Questionnaire. NC captures the tendency to think about one's thoughts as something that needs to be controlled and regulated at all times. (Example item: "*I should be in control of my thoughts all of the time"*).	6 – 24<br>Higher scores refer to <u>more need for control</u>.
PB	Positive beliefs about rumination	Summary score of the Positive Beliefs about Rumination Scale. PBRS measures the general tendency to interpret rumination as a positive and useful process that aims to solve the underlying problem. (Example item: "*I need to ruminate about my problems to find answers to my depression"*).	9 – 36<br>Higher scores refer to <u>more positive beliefs</u> about rumination.

NB-U	Negative beliefs about uncontrollability and harmfulness	Summary score of the factor Uncontrollability and Harmfulness of Rumination from the Negative Beliefs about Rumination Scale. NBUH measures the general tendency to consider rumination as harmful thinking that cannot be controlled. (Example item: "*How much do you agree that … ruminating is uncontrollable*").	8 – 32 Higher scores refer to more feelings of uncontrollability.
NB-S	Negative beliefs about social consequences	Summary score of the factor Negative Interpersonal and Social Consequences from the Negative Beliefs about Rumination Scale. NBSC measures the belief that rumination harms social interactions. (Example item: "*How much do you agree that … ruminating causes me to be rejected by others*").	5 – 20 Higher scores refer to a stronger belief about negative social consequences.

Table S2. Correlation Matrix with Key Variables

	PGF	EC	CC	PB	M-SC	M-NC	NB-U	NB-SC	PRO	RUM	DEP	PER
PGF		-.31	.18	.21	.16	.17	.26	.21	-.25	.33	.30	.13
EC			-.47	-.17	-.21	-.24	-.47	-.40	.43	-.55	-.50	-.26
CC				.21	.15	.32	.38	.34	-.32	.46	.41	.22
PB					.41	.36	.37	.26	-.17	.50	.38	.22
M-SC						.48	.43	.25	-.08	.50	.32	.32
M-NC							.43	.49	-.21	.46	.36	.35
NB-U								.66	-.40	.70	.63	.31
NB-SC									-.33	.53	.53	.36
PRO										-.45	-.60	-.02
RUM											.70	.37
DEP												.28
PER												

Notes. PGF – promotion goal failure, EC – effortful control, CC – cognitive confidence, PB – positive beliefs about rumination, M-SC – self-consciousness, M-NC – need for control, NB-U – negative beliefs about uncontrollability, NB – SC – negative beliefs about social consequences, PRO – promotion focus, RUM – rumination, DEP – depression, PER – perfectionism. All correlations in *italic* were statistically significant at $p < .005$ except PER – PRO ($p > 0.6$), and M-SC – PRO ($p > 0.08$). P-values were calculated with *rcorr()* from *Hmisc* package.

Table S3. Edge Weight Matrix for the GGM

	PGF	EC	CC	PB	M-S	M-N	NB-U	NB-S	PRO	RUM	DEP	PER
PGF	.00	-.13	.00	.00	.00	.00	.00	.00	.00	.00	.00	.00
EC		.00	-.24	.00	.00	.00	.00	.00	.16	-.21	.00	.00
CC			.00	.00	.00	.15	.00	.00	.00	.14	.00	.00
PB				.00	.16	.00	.00	.00	.00	.25	.00	.00
M-S					.00	.30	.15	-.15	.13	.22	.00	.00
M-N						.00	.00	.28	.00	.00	.00	.00
NB-U							.00	.41	.00	.27	.16	.00
NB-S								.00	.00	.00	.12	.14
PRO									.00	.00	-.40	.19
RUM										.00	.27	.10
DEP											.00	.00
PER												.00

Notes. PGF – promotion goal failure, EC – effortful control, CC – cognitive confidence, PB – positive beliefs about rumination, M-SC – self-consciousness, M-NC – need for control, NB-U – negative beliefs about uncontrollability, NB – SC – negative beliefs about social consequences, PRO – promotion focus, RUM – rumination, DEP – depression, PER - perfectionism

GGM Accuracy and Stability Checks:

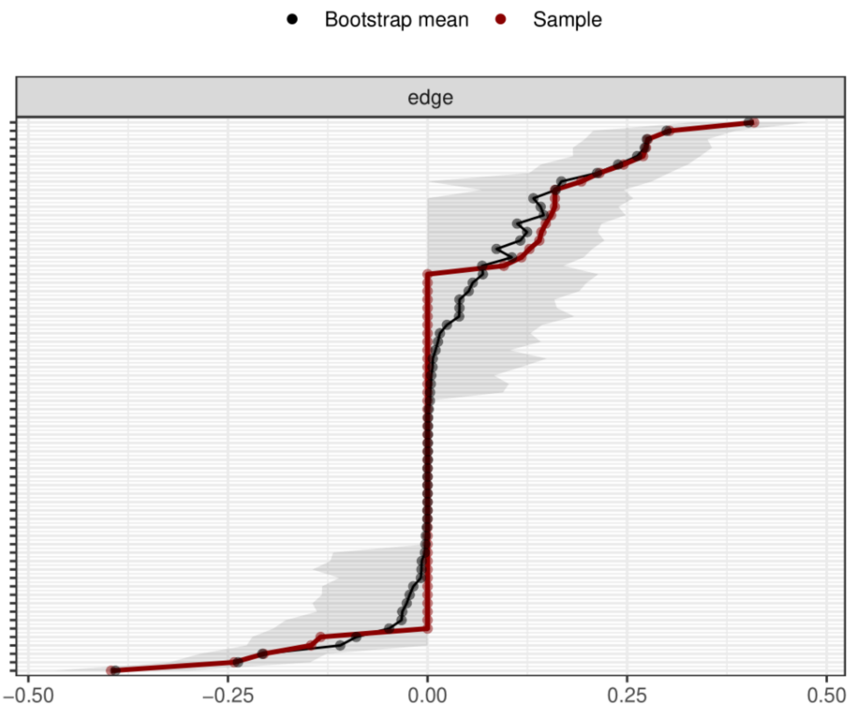

Figure S1. Accuracy: Bootstrapped edge weights. How often each edge in 1000 bootstraps was present? Red line indicates sample values, and the black line indicates mean of all bootstraps. The area indicates the bootstrapped confidence intervals. Nodes are not presented on the y-axes to avoid clutter.

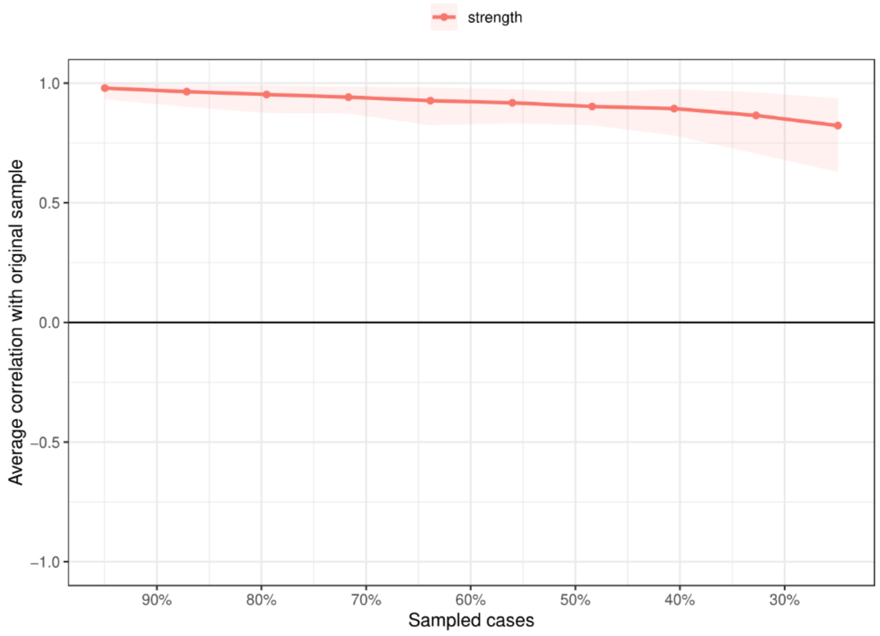

Figure S2. Stability: Case-drop bootstrap procedure. How stable is the order of the nodes in terms of strength? Line indicates the mean, and the area indicates the range from 2.5th to 97.5th quantile.

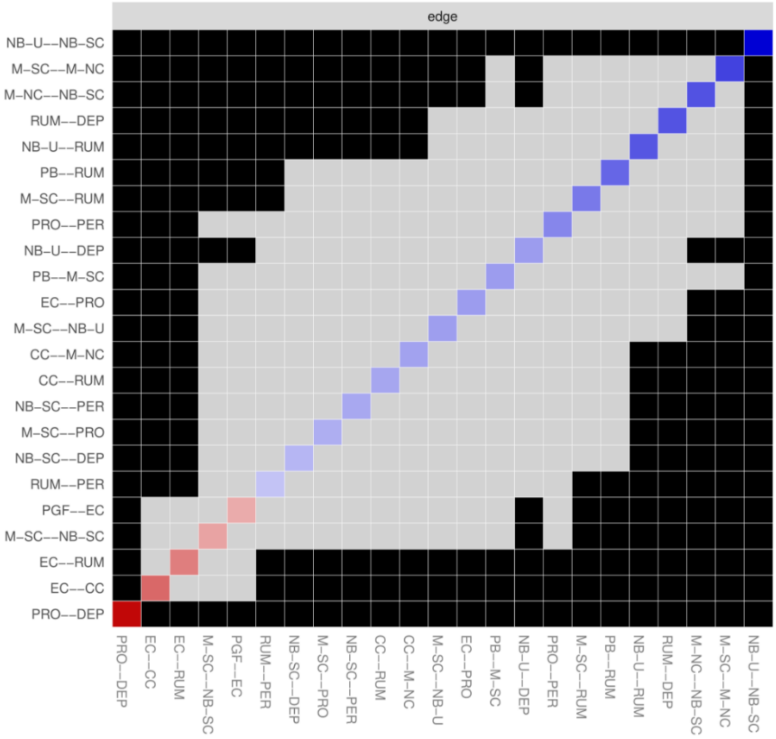

(A)

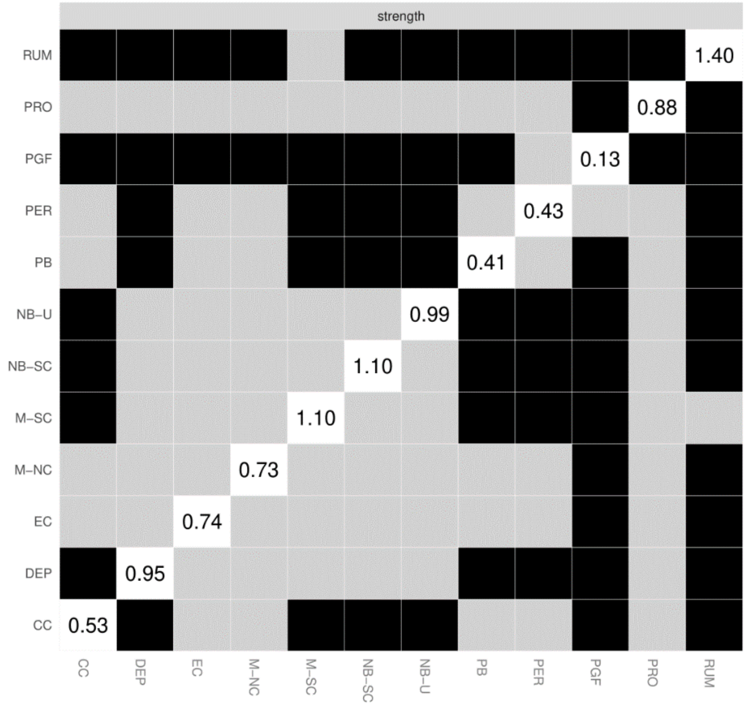

(B)

Figure S3. Bootstrapped difference tests between all non-zero edges (A) and nodes (B). Black squares indicate significant differences ($p<0.05$), and gray squares indicate nonsignificant differences between edges (A) or nodes (B).

Table S4. Regression Coefficients for the Directed Effects in the Averaged DAG (Figure 2A)

From (Parent)	To (Child)	Regression Coefficient
CC	EC	*-0.38*
PRO	EC	0.32
PB	M-SC	0.42
M-SC	M-NC	0.47
M-NC	NB-SC	0.22
NB-U	NB-SC	0.48
RUM	NB-U	0.67
EC	RUM	*-0.44*
PB	RUM	0.32
M-SC	RUM	0.28
NB-U	DEP	0.46
PRO	DEP	*-0.39*

Note. This table contains the regression coefficients for nodes in the averaged DAG. Positive values indicate that an increase in the parent node will increase the value of the child node, and negative values indicate that an increase in the parent node will decrease the value of the child node and vice versa. These values are indicative of the valence of potential causal effects. Negative effects are formatted in *italic.*

Table S5. PC Stable DAG Bootstrap Results

From	To	Strength	Direction
PGF	EC	.785	.555
PGF	CC	.008	.500
PGF	PB	.169	.621
PGF	M-SC	.006	.833
PGF	M-NC	.010	.900
PGF	NB-U	.009	.556
PGF	NB-SC	.032	.906
PGF	PRO	.223	.558
PGF	RUM	.390	.904
PGF	DEP	.115	.800
PGF	PER	.011	.500
EC	PGF	.785	.445
EC	CC	1.000	.472
EC	PB	.000	.000
EC	M-SC	.000	.000
EC	M-NC	.000	.000
EC	NB-U	.169	.322
EC	NB-SC	.206	.570
EC	PRO	.938	.254
EC	RUM	.980	.751
EC	DEP	.214	.685
EC	PER	.128	.582
CC	PGF	.008	.500
CC	EC	1.000	.529
CC	PB	.000	.000
CC	M-SC	.000	.000
CC	M-NC	.592	.684
CC	NB-U	.025	.460
CC	NB-SC	.057	.702
CC	PRO	.130	.458
CC	RUM	.752	.868
CC	DEP	.201	.662
CC	PER	.056	.696
PB	PGF	.169	.379
PB	EC	.000	.000
PB	CC	.000	.000
PB	M-SC	.940	.602
PB	M-NC	.579	.623
PB	NB-U	.013	.385
PB	NB-SC	.000	.000
PB	PRO	.000	.000
PB	RUM	.991	.798
PB	DEP	.126	.679
PB	PER	.015	.633

M-SC	PGF	.006	.167
M-SC	EC	.000	.000
M-SC	CC	.000	.000
M-SC	PB	.940	.398
M-SC	M-NC	1.000	.555
M-SC	NB-U	.421	.277
M-SC	NB-SC	.000	.000
M-SC	PRO	.000	.000
M-SC	RUM	.939	.909
M-SC	DEP	.000	.000
M-SC	PER	.623	.467
M-NC	PGF	.010	.100
M-NC	EC	.000	.000
M-NC	CC	.592	.316
M-NC	PB	.579	.377
M-NC	M-SC	1.000	.446
M-NC	NB-U	.000	.000
M-NC	NB-SC	1.000	.517
M-NC	PRO	.003	.667
M-NC	RUM	.073	.596
M-NC	DEP	.000	.000
M-NC	PER	.616	.416
NB-U	PGF	.009	.444
NB-U	EC	.169	.678
NB-U	CC	.025	.540
NB-U	PB	.013	.615
NB-U	M-SC	.421	.723
NB-U	M-NC	.000	.000
NB-U	NB-SC	1.000	.527
NB-U	PRO	.012	.500
NB-U	RUM	1.000	.456
NB-U	DEP	.922	.593
NB-U	PER	.002	.500
NB-SC	PGF	.032	.094
NB-SC	EC	.206	.430
NB-SC	CC	.057	.298
NB-SC	PB	.000	.000
NB-SC	M-SC	.000	.000
NB-SC	M-NC	1.000	.484
NB-SC	NB-U	1.000	.474
NB-SC	PRO	.001	1.000
NB-SC	RUM	.003	1.000
NB-SC	DEP	.781	.522
NB-SC	PER	.793	.458
PRO	PGF	.223	.442
PRO	EC	.938	.746
PRO	CC	.130	.542
PRO	PB	.000	.000

PRO	M-SC	.000	.000
PRO	M-NC	.003	.333
PRO	NB-U	.012	.500
PRO	NB-SC	.001	.000
PRO	RUM	.003	.500
PRO	DEP	1.000	.518
PRO	PER	.009	.000
RUM	PGF	.390	.096
RUM	EC	.980	.249
RUM	CC	.752	.132
RUM	PB	.991	.202
RUM	M-SC	.939	.091
RUM	M-NC	.073	.404
RUM	NB-U	1.000	.545
RUM	NB-SC	.003	.000
RUM	PRO	.003	.500
RUM	DEP	1.000	.451
RUM	PER	.450	.376
DEP	PGF	.115	.200
DEP	EC	.214	.315
DEP	CC	.201	.338
DEP	PB	.126	.321
DEP	M-SC	.000	.000
DEP	M-NC	.000	.000
DEP	NB-U	.922	.407
DEP	NB-SC	.781	.478
DEP	PRO	1.000	.483
DEP	RUM	1.000	.549
DEP	PER	.005	.600
PER	PGF	.011	.500
PER	EC	.128	.418
PER	CC	.056	.304
PER	PB	.015	.367
PER	M-SC	.623	.533
PER	M-NC	.616	.584
PER	NB-U	.002	.500
PER	NB-SC	.793	.542
PER	PRO	.009	1.000
PER	RUM	.450	.624
PER	DEP	.005	.400

Averaged DAG (MMHC):

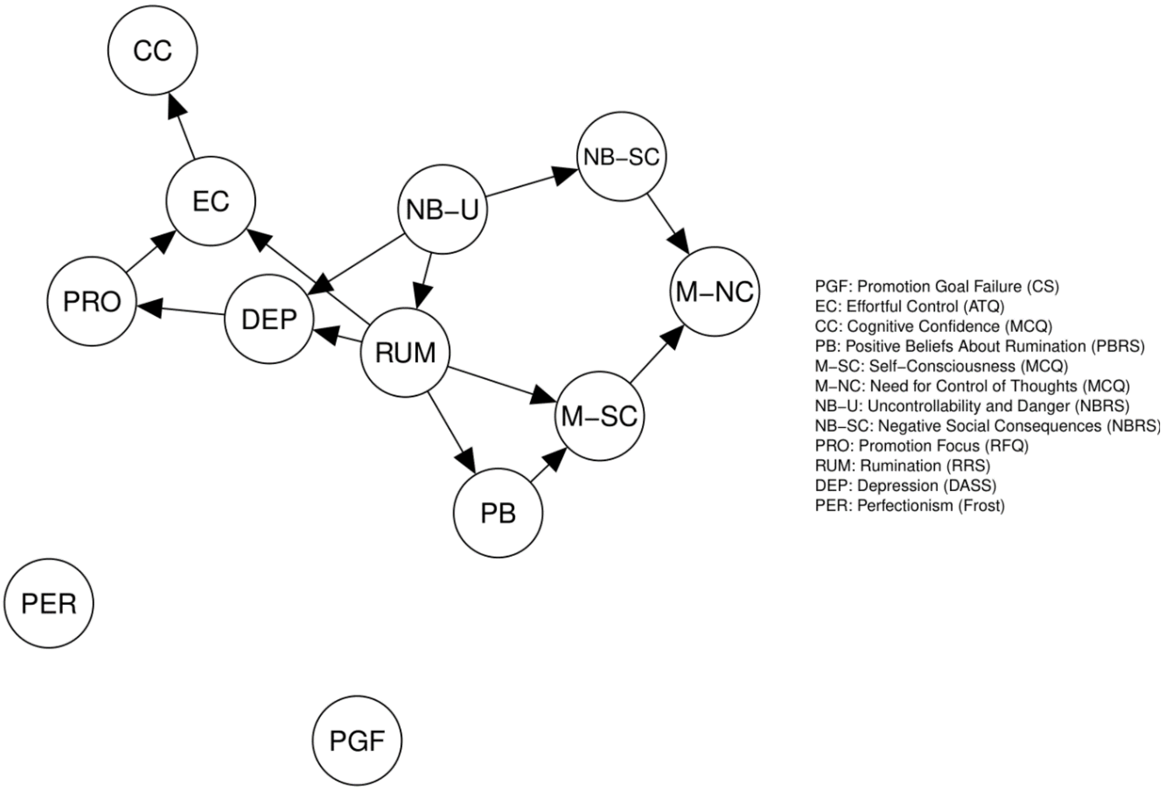

Figure S4. Averaged DAG from 1000 bootstrapped models obtained with the MMHC algorithm

Table S6. Sample age, sex, and ethnicity distributions

	Women					Men				
Age (years)	18-27	28-37	38-47	48-57	58+	18-27	28-37	38-47	48-57	58+
Asian	4	4	3	2	3	6	5	4	1	3
Black	3	2	2	1	1	2	3	2	1	0
Mixed	1	1	2	0	1	1	2	1	1	1
Other	0	1	0	0	0	1	0	1	0	0
White	35	33	38	38	80	33	29	39	35	66

Note. 6 subjects selected “other” or “prefer not to respond”. This distribution corresponds to the UK census data.
